# Polyphenolics from *Albizia harveyi* Exhibit Antioxidant Activities and Counteract Oxidative Damage and Ultra-Structural Changes of Cryopreserved Bull Semen

**DOI:** 10.3390/molecules22111993

**Published:** 2017-11-17

**Authors:** Mansour Sobeh, Soha A. Hassan, Mohamed A. El Raey, Wael A. Khalil, Mahmoud A. E. Hassan, Michael Wink

**Affiliations:** 1 Institute of Pharmacy and Molecular Biotechnology, Heidelberg University, Im Neuenheimer Feld 364, 69120 Heidelberg, Germany; 2 Department of Basic Sciences, Faculty of Dentistry, October 6 University, Cairo 12566, Egypt; sohaahmed1999@yahoo.com; 3 Department of Phytochemistry and Plant Systematics, National Research Center, Dokki, Cairo 12622, Egypt; elraiy@gmail.com; 4 Department of Animal Production, Faculty of Agriculture, Mansoura University, Mansoura 35516, Egypt; waelahmedk@gmail.com; 5 Animal Production Research Institute, Dokki, Giza 12619, Egypt; m.hassan55213@gmail.com

**Keywords:** *Albizia harveyi*, polyphenols, HPLC-PDA-ESI-MS/MS, antioxidant, sperm ultra-structure, cryopreservation

## Abstract

*Albizia harveyi* is a tropical deciduous tree, found across South and Eastern Africa and widely used in traditional medicine. The leaf extract ameliorated the damaging effects of the frozen-thawing process in cryopreserved bull semen. In a dose-dependent pattern, sperm motility, viability, and membrane integrity were improved compared to the untreated control. Furthermore, the extract increased the percentage of viable sperm cells and reduced the percentages of early apoptotic and apoptotic sperm cells as well as the damage in sperm ultra-structure. These activities are in agreement with the robust antioxidant properties in vitro and in the seminal fluid as observed in the total antioxidant capacity and the lipid peroxidation parameter malondialdehyde. LC-MS yielded 35 compounds. The extract was dominated by quercetin-*O*-galloyl-hexoside and quercetin-*O*-pentoside, along with other flavonoid glycosides. The polyphenols are probably responsible for the observed activities. In conclusion, the current findings show that *A. harveyi* leaves are rich in bioactive polyphenols with functional properties, validating its traditional use.

## 1. Introduction

Reactive oxygen species (ROS) are very reactive and unstable compounds with one or more unpaired electrons in their outer shells. They are produced continuously by normal metabolic processes within all biological systems. Elevated ROS production, caused by stress, radiation and pollution, can elicit cellular and metabolic disturbances including lipid peroxidation, protein oxidation, tissue damage, and DNA mutations, which may be implicated in the pathology of several health disorders such as cancer, low immune functions, liver injury, cardiovascular diseases, and inflammation. ROS could be also involved in the development of ageing and neurodegenerative diseases as well the etiology of male infertility [1].

Within biological systems, several chemical scavengers, endogenous enzymes (glutathione peroxidase, catalase, superoxide dismutase), antioxidants and vitamins (ascorbic acid, α-tocopherol, β-carotene) are able to quench the excess of free radicals formed in cells and thus delay or restrict the damage that ROS can do to the cell and/or the cellular components. Plants, which produce more than 4000 phenolic and polyphenolic compounds are also considered as good antioxidants and still considered as an important source for the development of novel pharmacological agents [2,3].

*Albizia harveyi* (Fabaceae) is essentially rich in proanthocyanidins, among other phytochemicals, and is widely used in traditional medicine. The genus comprises approximately 130 species and they are widespread along Africa and Central and South America. *A. harveyi* is used in traditional African medicine to treat a number of conditions, including stomachaches, hypertension, convulsions, intestinal worms, chest pains, and wounds [4].

A wide variety of secondary metabolites has been reported from this genus, including saponins, flavonoids, alkaloids, and triterpenes. For instance, we have recently characterized 39 compounds in the methanol extract of *A. harveyi* bark and the majority of them were flavan-3-ol derivatives such as (epi)catechin, (epi)gallocatechin, and condensed tannins including (epi)catechin dimers, trimers, and tetramers [4]. A wide variety of pharmacological properties have also been identified including antitumor, antioxidant, hepatoprotective, antidiabetic, and sedative activities [4,5,6].

In this investigation, the polyphenolic constituents of a bioactive methanol extract of *A. harveyi* leaves were comprehensively characterized by HPLC-PDA-MS/MS. We also studied the antioxidant activities in vitro. Furthermore, we investigated the effect of supplementation of the leaf extract for the cryopreservation of bull sperm. Sperm characteristics, membrane integrity, chromatin damage, apoptosis, sperm ultra-structure, total antioxidant and lipid peroxidation were determined after cryopreservation.

## 2. Results

### 2.1. Phytochemical Profiling of A. harveyi Leaf Extract

**Figure 1:**
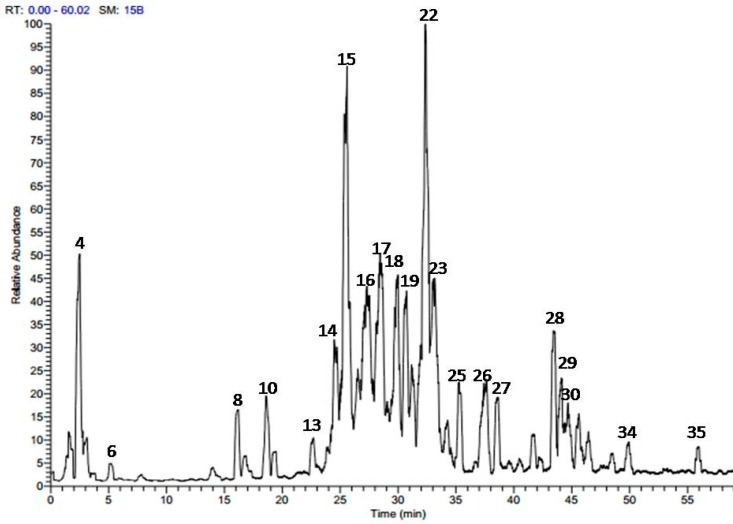
Total ion chromatogram of the methanol extract of *A. harveyi* leaves [LC-MS-ESI (−)].

The methanol extract obtained from *A. harveyi* leaves was analyzed by online combination of HPLC-PDA-ESI-MS/MS. The analysis revealed a total of 35 secondary metabolites. Figure 1 presents the LC-MS base peaks in the negative ionization mode ESI (−) and the identified peaks are listed in Table 1.

A series of myricetin, quercetin, and kaempferol derivatives was found to be the predominant class of compounds in the extract, along with some phenolic acids such as gallic acid, ferulic acid as well as their derivatives (Table 1). Different pattern was reported for the bark where condensed tannins dominated [4].

Ferulic acid was detected with [M − H]^−^ at *m*/*z* 193 and a main fragment at *m*/*z* 147 while another peak with [M − H]^−^ at *m*/*z* 169 with a daughter ion at *m*/*z* 125 was assigned to gallic acid [7,8,9]. Two peaks showed a molecular ion peak [M − H]^−^ at *m*/*z* 337 and a daughter ion peak at *m*/*z* 191; they were assigned to 3-*p*-coumaroylquinic acid, 4-*O*-*p*-coumaroylquinic acid, as previously reported [9].

A series of peaks showed molecular ion peaks [M − H]^−^ at *m*/*z* 433, 447, 463, 609, and 615 and a daughter ion at *m*/*z* 301; they were tentatively identified as quercetin-*O*-pentoside [M − H—132], quercetin-*O*-rhamnoside [M − H—146, loss of rhamnose], quercetin-*O*-glucoside [M − H—162, loss of glucose], quercetin-*O*-coumaroyl-hexoside [M − H—146–162], and quercetin-*O*-galloyl-hexoside [M − H—152–162], respectively [8,11,12]. A precursor with [M − H]^−^ at *m*/*z* 639 and fragments at *m*/*z* 463 [M − H—176, loss of feruloyl moiety] and 301 [M − H—176–162, loss of feruloyl-hexoside] confirmed the presence of quercetin-*O*-feruloyl-hexoside while the precursor at [M − H]^−^ *m*/*z* 629 and two daughter ions at *m*/*z* 463 and 301 were assigned to quercetin methyl galloyl-hexoside. Also, the quercetin aglycone was detected at [M − H]^−^ *m*/*z* 301 and two fragments ions 151, and 179 (Table 1).

Several peaks were also detected with [M − H]^−^ at *m*/*z* 417, 447, 593, 599 and a daughter ion at *m*/*z* 285; they were assigned to kaempferol-*O*-pentoside [M − H—132, loss of pentose moiety], kaempferol-*O*-hexoside [M − H—162], Kaempferol-*O*-coumaroyl-hexoside [M − H—146–162], and kaempferol-*O*-galloyl-hexoside [M – H—152–162], respectively along with the kaempferol aglycone at *m*/*z* 285 [8,13]. Other peaks showed a molecular ion peak [M − H]^−^ at *m*/*z* 449, 479 and a main fragment at 317; they were characterized as myricetin-*O*-pentoside and myricetin-*O*-hexoside as described in [10] (see Table 1).

It is worth highlighting a precursor that showed [M − H]^−^ at *m*/*z* 385 and a daughter ion at *m*/*z* 223 [M – H—162]^−^ which confirmed the presence of sinapic acid-*O*-hexoside. A precursor with [M − H]^−^ at *m*/*z* 299 and a main fragment at *m*/*z* 153 [M – H—146, loss of rhamnose moiety] confirmed the presence of gentisic acid-*O*-rhamnoside, (see Table 1).

### 2.2. Biological Activities

#### 2.2.1. Total Phenolic Content and Antioxidant Activities In Vitro

In vitro testing of the antioxidant activities of *A. harveyi* leaf extract in DPPH and FRAP assays revealed promising activities compared to the positive control Epigallocatechin gallate (EGCG). These activities can be attributed to the high total phenolic content as investigated using the Folin-Ciocalteu method and amounted 371 mg gallic acid equivalent/g extract (see Table 2).

#### 2.2.2. Antioxidant Activities in Bull Semen Cryopreservation

Semen cryopreservation is a key procedure that facilitates assisted reproductive technology applications for both humans and animals. Several factors affect the quality of semen cryopreservation including chemical, physical, osmotic and oxidative stresses, type of extenders and cryoprotectants, and cooling and thawing rates. Excessive ROS production and the consequent oxidative stress have a major detrimental impact on semen quality during freezing and thawing of cryogenic vials and can decrease sperm motility, increase DNA damage and cell apoptosis, and disturb the plasma membrane functions, resulting in reduce sperm-fertilization capacity [18]. Some plants were able to counteract these deleterious effects due to their potential antioxidant activities [19].

##### Quantification of Oxidative Stress Markers in Seminal Plasma of Post-Thawed Bull Semen

To monitor the antioxidant activities of the studied extract in the seminal plasma, we investigated the total antioxidant capacity (TAC) and lipid peroxidation (MDA). Pre-treatment of the sperm with the extract significantly increased TAC and diminished MDA in a concentration dependent manner compared to the control group (see Table 3).

##### Effect of the Extract on Post-Thawing Sperm Characteristics

To study the effect of our extract on sperm characteristics in post-thawed bull semen, the sperm cells were frozen with three different extract concentrations (0.5, 1.0 and 1.5 µg/mL), kept at −196 °C and then the samples were thawed. Sperm motility, viability, and membrane integrity were significantly improved in the treated samples in a concentration dependent pattern, compared to the control. Also, the percentage of sperm abnormality and sperm chromatin damage was significantly reduced at a concentration of 1.5 µg/mL (*p* < 0.05), (see Table 4).

##### Anti-Apoptosis Effects

The annexin-V binding assay is a powerful marker in detecting changes in the sperm plasma membrane that occur during the process of apoptosis [20]. In apoptosis, phosphatidylserines are located outside the plasma membrane of sperm and then can be detected by fluorescein isothiocyanate (FITC)-conjugated annexin-V. The annexin-V binding assay describes four sperm subpopulations in bull semen samples, which come in agreement with results revealed by electron microscopy: two categories of live spermatozoa (i) viable (A−/PI−) with membrane intact and (ii) early apoptotic spermatozoa (A+/PI−) with minimal membrane changes; and two other categories of dead spermatozoa (i) apoptotic cells (A+/PI+) which have damaged membranes (swelling membrane); and (ii) necrotic cells (A−/PI+) with completely lost sperm membrane integrity as well as chromatin changes [21].

**Figure 2:**
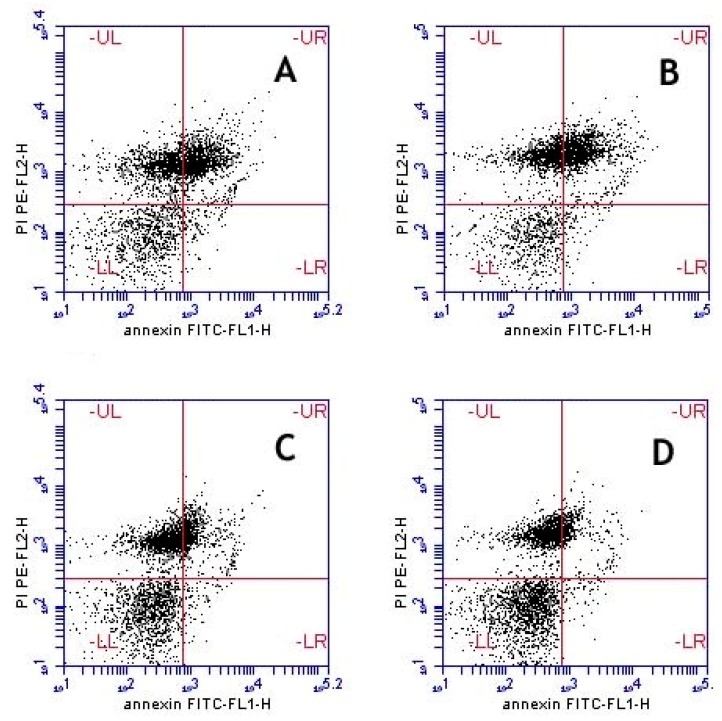
Flow cytometry contour plots of frozen/thawed spermatozoa labelled with annexin V-fluorescein isothiocyanate (FITC) fluorescence and propidium iodide (PI) fluorescence. (**A**) Control; (**B**) *A. harveyi* leaf extract 0.5 µg/mL; (**C**) *A. harveyi* leaf extract 1 µg/mL; (**D**) Leaf extract 1.5 µg/mL. In each graph, the upper right quadrant (UR) represents apoptotic spermatozoa binding annexin-V and PI (A+/PI+). The upper left quadrant (UL) represents necrotic cells excluding annexin-V and binding PI (A−/PI+). The lower left quadrant (LL) contains viable spermatozoa, which are negative for annexin-V and exclude PI staining (A−/PI−). The lower right quadrant (LR) shows early apoptotic spermatozoa, which bind annexin-V but exclude PI (A+/PI−).

In the present study, a high percentage of apoptotic sperm cells and a low percentage of viable spermatozoa were recorded after freeze-thawing process. Treatment of semen extender with the leaf extract significantly improved the percentage of viable sperm and decreased the percentage of early apoptotic and apoptotic sperm cells in a dose dependent fashion compared to the control (Table 5). On the other hand, the percentage of necrotic sperm was not affected. Representative figures are shown for each corresponding group in Figure 2.

##### Effect of the Extract on Sperm Ultra-structure Post Thawing

Following the freezing−thawing process, the cell membrane of the negative control group showed many changes ranging from discontinuity until complete damage. Also, an apical ridge formed on the tip of acrosomal cap and detached from the nuclear membrane leaving a sub- acrosomal space. Small vesiculations also appeared and the membrane was swallowed and may be finally degenerated.

**Figure 3:**
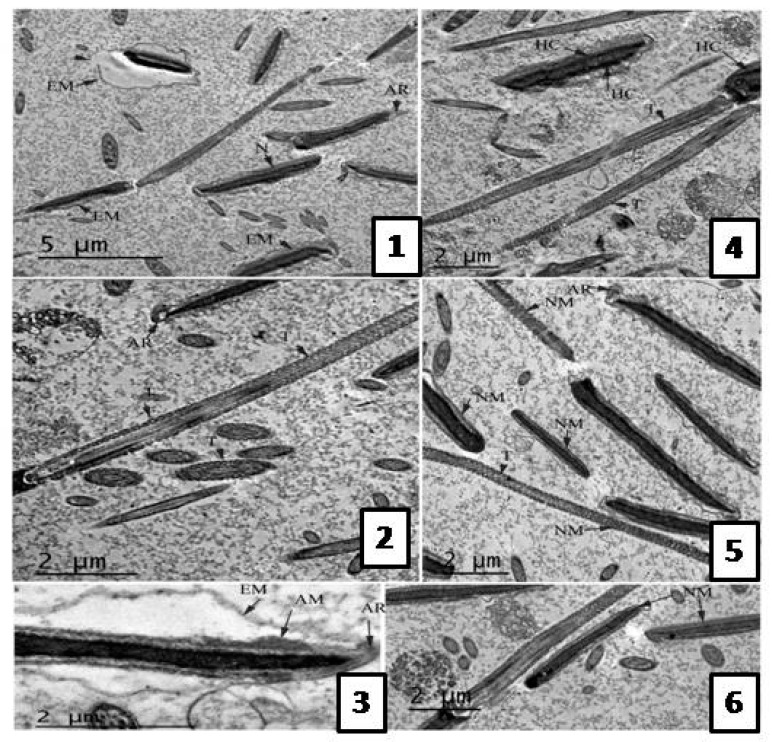
TEM photomicrographs of bull sperm head in groups treated with *A. harveyi* extract; (**1**–**3**) TEM photomicrographs treated with 0.5 mg/mL. Sagital sections for acrosomal region and mid piece showing extension of cell membrane (EM) with different degrees. Discontinuous membrane (DC) also observed. (**2**) Sagital section for neck region representing electron translucent (T) parts of mitochondrial sheath and unequal distribution. (**3**) Sagital section for acrosomal cap showing detached outer plasma membrane, diffusion of acrosomal material (AM) into subacrosomal space and apical ridge (AR) formation; (**4**–**6**) TEM photomicrographs treated with 1.0 mg/mL extract. (**4**) Sagital section from neck region showing heterogenous chromatin (HC) condensation in acrosomal region. (**5**,**6**) Different sagital sections from acrosom and neck region representing mild extension of cell membrane (EM) and apical ridge formation (AR). Also, mild degree of mitochondrial sheath damage was observed and some regions have translucent mitochondria while other regions have normal mitochondria (NM).

**Figure 4:**
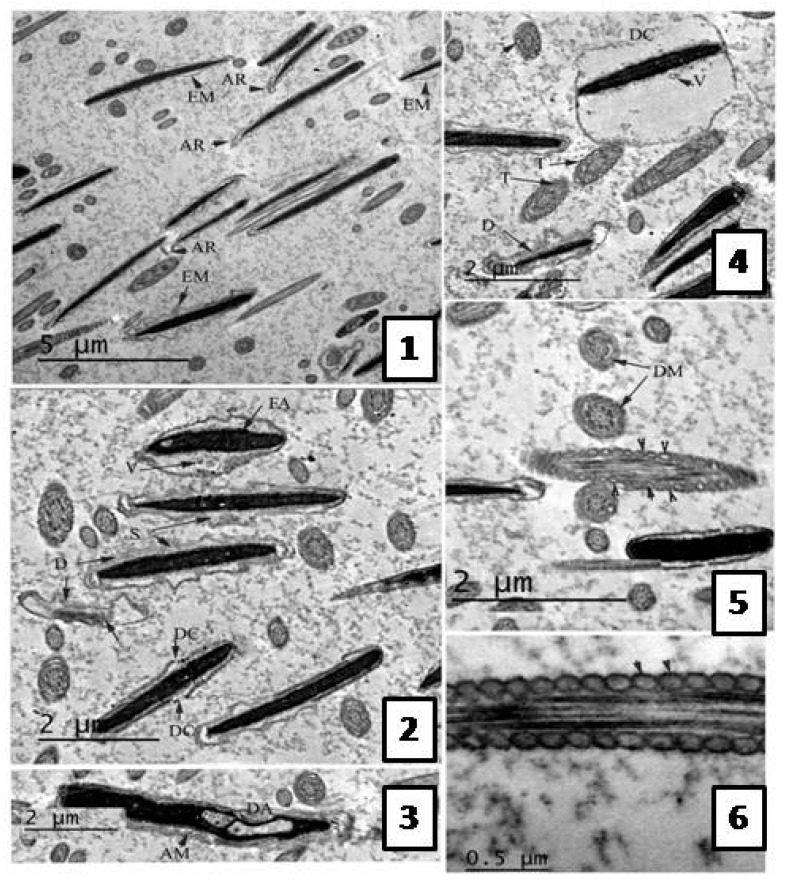
TEM photomicrographs of sagital sections of the bull sperm in control group representing different stages of damage after freezing—thawing process. (**1**) The plasma membrane is extended (EM) away from nucleus and apical ridge (AR) is formed near the tip of acrosomal cap (AC). (**2**) The plasma membrane at acrosomal region and sub acrosomal region detached from the nuclear envelop leaving sub-membranous swelling (S) and vesiculations (V) appears inside degenerated cell membrane (D). Discontinuous membrane (DC) is also noted in addition to flat acrosome (FA). (**3**) The section revealed a marked damage of acrosomal cap (DA), cell membrane and diffusion of acrosomal dense material (AD) into the previously formed space. (**4** and **5**) Different sections of neck region show abnormal mitochondrial sheath. Unequal size and distribution (arrow heads), electron translucent (T) and degenerated mitochondria (DM) were also detected. (**6**) Longitudinal section for mitochondrial sheath show amorphous material of mitochondria (arrow heads), absence of cristae and cell membrane.

**Figure 5:**
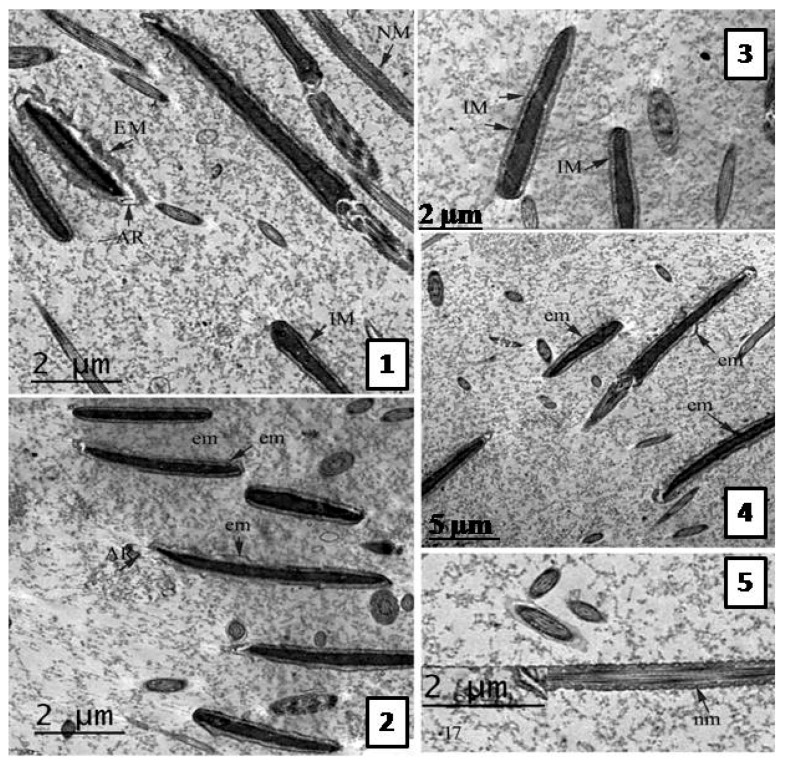
TEM photomicrographs of bull sperm head in groups treated with 1.5 µg *A. harveyi* extract. (**1**–**5**) Different sagital sections representing cell membrane of acrosomal cap with intact plasma membrane (IM) in many sperms of the same section. Early degree of cell membrane extension also was found (EM). The electron dense mitochondria is regularly placed in both transverse and longitudinal sections.

The electron dense material of acrosom also diffused into those vesiculations, see Figure 3. The group treated with 0.5 µg/mL *A. harveyi* extract showed abnormalities similar to the control, see Figure 4. However, the second group (1.0 µg/mL extract) exhibited moderate changes observed by early damage stages (see Figure 3) while the third treated group which was supplemented with 1.5 µg/mL of the extract showed limited changes (Figure 5).

## 3. Discussion

In the present study, we investigated the antioxidant activities in vitro and in semen-sperm based model. The studied extract exhibited pronounced antioxidant activities in vitro. Our results are in a comparable magnitude as described before for other crude plant extracts from the genus *Albizia* [4,22].

Cryopreservation and freeze-thawing processes are associated with ROS production, DNA fragmentation and apoptosis in sperm cells [23]. Also, sperm plasma membrane is rich in polyunsaturated fatty acids and this makes it prone to peroxidation [24]. In this study, detrimental effects were observed in the control group (untreated control). Treatment of the sperm with *A. harveyi* extract showed positive effects on sperm parameters (progressive motility, viability, abnormality, membrane integrity, chromatin damage, apoptosis and ultra-structure). Also, the total antioxidant capacity and lipid peroxidation in seminal plasma of post-thawing were observed. These activities might be attributed to the presence of tannins, flavonoids, and phenolic acids in the studied extract. Several medicinal plant extracts such as from *Rosmarinus officinalis*, *Moringa oleifera,* and *Arctium lappa* roots showed similar properties [19,25,26]. Also, *Salvia officinalis* extracts exhibited comparable activities and improved the sperm quality and protected spermatozoa in a concentration dependent fashion against oxidative stress damage as a result of cryopreservation [27]. Additionally, Malo et al., reported that antioxidants from Fennel (*Foeniculum vulgare*) exerted a protective effect on the plasma membrane and improved sperm motility [28]. Other extracts including green tea and strawberry fruit (*Arbutus unedo*) as well as individual compounds such as quercetin, resveratrol, tocopherol, and ascorbic acid observed promising activities [29,30]. In summary, an overall improvement after freezing and post-thawing in the sperm parameters were observed, when sperm was treated with *A. harveyi* leaf extract.

## 4. Materials and Methods

### 4.1. Extraction

*A. harveyi* leaves were collected from trees in Lupaga Site in Shinyanga, Tanzania. A plant sample is kept at IPMB, Heidelberg University under accession number P7286. At room temperature, plant leaves were dried, ground and extracted with methanol for 3 days (6 × 500 mL, maceration extraction). The extracts were then combined, filtered, reduced under vacuum at 40 °C giving a semisolid residue. The latter was frozen and then subjected to lyophilization giving fine dried powder (10%).

### 4.2. HPLC-PDA-MS/MS

The extract was analyzed by HPLC-PDA-MS/MS using a ThermoFinniganLC system (ThermoElectron Corporation, Austin, TX, USA) [31]. A Zorbax Eclipse XDB-C18, Rapid resolution, 4.6 × 150 mm, 3.5 µm column was used (Agilent, Santa Clara, CA, USA). A gradient consists of water and acetonitrile (ACN), each having 0.1% formic acid, was applied and acetonitrile was increased from 5 to 30% within 60 min in 1 mL/min flow rate and a 1:1 split before the ESI source. The sample was injected using autosampler. LCQ-Duo ion trap having a ThermoQuest ESI source was used for MS analysis. Xcalibur software (Xcalibur™ 2.0.7, Thermo Scientific, Waltham, MA, USA) was used to control the system. MS operating parameters in the negative mode were used as described in [7].

### 4.3. Antioxidant Activity in Vitro

The Folin-Ciocalteu method was used to quantify total phenols [32]. Radical scavenging activity (DPPH assay) and ferric reducing antioxidant power (FRAP assay) were applied to assess the antioxidant activities. Assays were done following the previously described protocols [33].

### 4.4. Bull Semen Cryopreservation

#### 4.4.1. Collection and Selection of Semen Samples

Ejaculates were collected from five healthy, fertile Friesian bulls, 4–8 year old, raised at the international livestock management training center, Skha, Kafr El-Sheikh, Egypt. Semen was collected twice a week for 3 weeks. The bulls were kept under standard conditions of feeding and management. Semen was collected by artificial vagina (Neustadt/Aisch, Müller, Nürnberg, Germany) pre-warmed to 42 °C. The percentage of progressive motility for each sample was determined subjectively by two experienced researchers using a phase contrast microscope with 200× magnification. Ejaculates with ≥70% motility were selected for cryopreservation experiments.

#### 4.4.2. Cryopreservation Procedures

Semen was cryopreserved using standard production procedures in our AI centers according to Chen et al., [34] with some modifications. Briefly, semen was gradually diluted at 37 °C with tris-yolk fructose (TYF) extender containing 3.028 g/dL trisaminomethane, 1.675 g/dL citric acid anhydrous, 1.25 g/dL fructose, 7% (*v*/*v*) glycerol, 20% (*v*/*v*) egg yolk, 100 IU/mL penicillin, and 100 µg/mL streptomycin. The extension rate was semen: extender (1:20). Diluted semen samples were kept at 4 °C in a cooling chamber for 4 h as an equilibration period then automatically filled in 0.25 mL French straws (IVM Technologies, L’Aigle, France), placed 4 cm above liquid nitrogen for 10 min then frozen in liquid nitrogen (−196 °C) as described by [35]. Samples were evaluated after thawing (37 °C for 30 s in water bath).

#### 4.4.3. Experimental Design

Pooled bull semen was extended with tris-yolk fructose extender supplemented with different concentration from *A. harveyi* extract (0.5, 1.0 and 1.5 µg/mL extender). Semen straws were stored in liquid nitrogen (−196 °C) for a month and then samples were thawed and evaluated as follow.

##### Assessment of Sperm Progressive Motility

Percentage of progressive sperm motility in each semen sample (10 µL diluted semen) was determined using a phase contrast microscope (DM 500, Leica, Switzerland) supplied with a hot stage adjusted to 37 °C.

##### Assessment of Sperm Viability and Abnormalities

A smear from diluted semen was made on a glass slide and was stained by eosin (1.67%) and nigrosin (10%) stain [36]. A total of 300 sperm were examined in each sample at 400× under light microscope (Leica DM 500). The number of dead spermatozoa (red stained) was counted. The number of sperm cells bearing head and tail morphological abnormalities were also recorded as previously described [37].

##### Determination of Membrane Integrity with Hypo-Osmotic Swelling Test

The hypo-osmotic swelling (HOS) test was used to evaluate the functional plasma membrane of spermatozoa as described by [38]. Briefly, 10 µL of semen was incubated with 100 µL hypo-osmotic solution (6.75 g/L fructose and 3.67 g/L sodium citrate, to give osmolality of 75 m Osmol/L) at 37 °C for 30 min. Afterwards, 10 µL of the mixture was placed on a micro-scope slide and covered with a cover slip. A total of 300 spermatozoa were evaluated and sperm with swollen and coiled tails were determined in each sample under phase-contrast microscopy (Leica DM 500) at 400×.

##### Determination of Chromatin Integrity with Toluidine Blue Staining

Toluidine blue staining was performed as previously described [39] with some modification. Smears obtained were fixed in ethanol-acetic acid (3:1, *v*/*v*) for 1 min and 70% ethanol for 3 min. Smears were hydrolyzed for 20 min in 4 mM hydrochloric acid, rinsed in distilled water and air-dried. One droplet of 0.025% toluidine blue in McIlvaine buffer (sodium citrate-phosphate) pH 4.0 was placed over each smear and then cover slipped. Smears were evaluated with light microscopy at 1000×. The percentage of chromatin damage was estimated by evaluating 300 sperm on each smear. Spermatozoa stained as green to light blue were considered to have normal chromatin while those stained dark blue to violet were considered to have damaged chromatin.

##### Fluorescent Staining of Sperm and Flow Cytometric Analysis

Semen samples were processed for annexin-V staining as described in [40] with some modifications. A total of 9 straws (each run 3 straws were previously cryopreserved from each treatment) were thawed for the flow cytometry (FC) analysis. Semen from each run was pooled together to avoid handling errors. Semen samples washed twice and then centrifuged (300 *g*, 10 min, 4 °C) with PBS and the supernatant was removed. The sperm pellet was re-suspended in binding buffer at a concentration of 1 × 10^6^ sperm/mL. 100 μL of sperm were transferred to a 5 mL culture tube with 5 μL of annexin-V (FITC label) and 5 μL PI (PI label), and incubated for 15 min in dark at room temperature (25 °C) and additional binding buffer (400 µL) was added to each tube. Flow cytometric evaluation was conducted within 5 min. Flow cytometric analyses were performed on Accuri C6 Cytometer (BD Biosciences, San Jose, CA, USA) using the Accuri C6 software (Becton Dickinson) for acquisition and analysis [41]. Platelet counting was done using the BD Accuri™ C6 flow cytometer. For the gated cells, the percentages of annexin-V negative or positive (A− or A+) and PI negative or positive (PI− or PI+) as well as double positive cells were evaluated.

##### Biochemical Assays in Seminal Plasma

Semen samples after thawing was centrifuged (2-16 KL, Sigma, Darmstadt, Germany) for 15 min at 1500 *g* at 4 °C, and then seminal plasma was separated and stored at −20 °C. Concentration of total antioxidant (TAC) [42] and malondialdehyde (MDA) [43] were analyzed by commercial kit (Biodiagnostic, Giza, Egypt) using spectrophotometer (SPECTRO UV-VIS AUTO, UV-2602, Labomed, Culver, CA, USA).

##### Transmission Electron Microscope (TEM) Evaluation of Semen Samples

Sperm samples were processed for TEM as described in [44] with some modifications. Briefly, samples (500 μL) were centrifuged and resuspended in a fixative solution composed of 4% glutaraldehyde in phosphate buffered saline for 2 h at 4 °C. Samples were then washed and post-fixed in 1% osmium tetroxide for 1 h at room temperature. Fixed samples were then dehydrated in an ethanol gradient, treated with propylene oxide and embedded in Epon resin (Epon 812; Electron microscopy Science, Hatfield, England)) and ultrathin-sectioned (60–70 nm) for TEM. Ultrathin sections were observed at 80 kV using a 2100 TEM (JEOL, Tokyo, Japan) at 80 KV. The sperm ultrastructure was examined in 300 spermatozoa per sample.

### 4.5 Statistical Analysis

Results were expressed as mean ± SEM. The statistical differences between the groups were determined using GraphPad Prism software (version 5, GraphPad Software, Inc., San Diego, CA, USA). One-way analysis of variance test (ANOVA) was applied followed by Tukey’s post hoc test to determine the statistical significance. A value of *p* < 0.05 was accepted as statistically significant.

## 5. Conclusions

LC-MS structural analysis of *A. harveyi* leaf extract resulted in the identification of 35 compounds. The extract is rich in flavonoids namely myricetin, quercetin, and kaempferol glycosides. The extract exhibited strong antioxidant activities in DPPH and FRAP assays as well as in cryopreserved bull semen against the deleterious effects of freezing-thawing process. *Albizia harveyi* is a promising plant with potential therapeutic properties and can be further explored in curing various diseases associated with oxidative stress and also for sperm preservation.

## Figures and Tables

**Table 1:** Chemical constituents of the methanol leaf extract of *A. harveyi.*

No.	Tentatively Identified Compounds	t_R_ (min.)	UV λmax	[M − H]^−^ (*m*/*z*)	MS/MS	Reference
**1**	Malic acid	1.37	-			[4]
**2**	Gallic acid	1.51	272	169	125	[7,8]
**3**	Ferulic acid	1.73	266, 307	193	147	[9]
**4**	Gentisic acid-*O*-rhamnoside	2.42	310	299	153	
**5**	3-*O-p*-Coumaroylquinic acid	3.15	276, 315	337	163, 191	[4]
**6**	4-*O*-*p*-Coumaroylquinic acid	5.20	281, 310	337	163, 191	[4]
**7**	Caffeic acid derivative	14.04	-	541	179, 389	
**8**	(*epi*)-Catechin-(*epi*)-catechin	16.75	277	577	289, 425	[4]
**9**	Sinapic acid-*O*-hexoside	17.16	265, 310	385	153, 223	
**10**	(epi)Catechin	18.70	280	289	179, 245	[4]
**11**	Lyoniresinol-*O*-hexoside	18.96	278	581	419	
**12**	Rosmarinic acid-*O*-hexoside	19.31	-	521	359	
**13**	Myricetin-*O*-hexoside	22.72	262, 357	479	317	[10]
**14**	Quercetin-*O*-galloyl-hexoside	24.61	265, 355	615	463, 301	[8,11]
**15**	Quercetin-*O*-galloyl-hexoside	25.75	267, 352	615	463, 301	[8,11]
**16**	Myricetin-*O*-pentoside	26.55	266, 360	449	317	[10]
**17**	Quercetin-*O*-galactoside	27.55	265, 357	463	301	[8,12]
**18**	Quercetin-*O*-glucoside	28.55	265, 355	463	301	[8,12]
**19**	Kaempferol-*O*-galloyl-hexoside	29.15	266, 352	599	447, 285	[8]
**20**	Quercetin-*O*-pentoside	29.98	265, 357	433	301	[12]
**21**	Kaempferol-*O*-hexoside	31.32	266, 352	447	151, 285	[8,13]
**22**	Quercetin-*O*-pentoside	32.45	265, 356	433	301	[12]
**23**	Quercetin-*O*-rhamnoside	33.33	265, 354	447	301	[14]
**24**	Kaempferol-*O*-pentoside	34.17	265, 350	417	285	[13]
**25**	Kaempferol-*O*-pentoside	35.26	265, 349	417	285	[13]
**26**	Quercetin-*O*-caffeoyl-hexoside	37.54	265, 335, 354	625	463, 301	[15]
**27**	Quercetin-*O*-caffeoyl-hexoside	38.67	269	625	463, 301	[15]
**28**	Quercetin-*O*-coumaroyl-hexoside	43.47	269, 280	609	463, 301	
**29**	Quercetin-*O*-coumaroyl-hexoside	44.12	268, 312	609	463, 301	
**30**	Quercetin-*O*-feruloyl-hexoside	44.80	270, 308	639	463, 301	[16]
**31**	Quercetin	45.56	265, 369	301	179, 151	[11,14]
**32**	Kaempferol-*O*-coumaroyl-hexoside	46.57	269, 315	593	447, 285	
**33**	Ferulic acid derivative	48.64	-	293	193, 236	
**34**	Quercetin methyl galloyl-hexoside	49.91	267, 303, 354	629	463, 301	
**35**	Kaempferol	55.84	268, 350	285	151, 285	[8,17]

**Table 2:** Antioxidant activities of the methanol extract of *A. harveyi* leaves.

Sample	Extract	EGCG (Epigallocatechin gallate)
DPPH [EC_50_, µg/mL]	16.3	3.5
FRAP [mM FeSO_4_/mg extract]	17.00	25

**Table 3:** Effect of *A. harveyi* leaf extract on total antioxidant capacity (TAC) and lipid peroxidation (malondialdehyde, MDA) in seminal plasma of bull frozen-thawed semen.

Sample	TAC (mM/L)	MDA (nmol/mL)
Untreated control	0.18 ± 0.00	67.26 ± 1.86
Extract 0.5 µg/mL	0.36 ± 0.10	56.76 ± 3.08 **
Extract 1.0 µg/mL	0.87 ± 0.04 ***	43.53 ± 0.84 ***
Extract 1.5 µg/mL	1.26 ± 0.16 ***	32.43 ± 0.99 ***

Results were expressed as mean ± SEM (*n* = 3) and significant differences were compared related to the control groups. ** *p* < 0.01 and *** *p* < 0.001 by One Way ANOVA and Tukey post hoctest.

**Table 4:** Effect of *A. harveyi* leaf extract on sperm characteristics of bull frozen-thawed semen.

Sample	Motility (%)	Viability (%)	Membrane Integrity (%)	Abnormality (%)	Chromatin Damage (%)
Untreated control	46.66 ± 1.76	46.66 ± 2.96	41.33 ± 1.76	27.33 ± 2.84	8.67 ± 1.76
Extract 0.5 µg/mL	51.55 ± 1.67	50.33 ± 2.90	46.66 ± 1.85	23.33 ± 3.92	7.33 ± 0.88
Extract 1.0 µg/mL	60.00 ± 2.88 ^¥^	58.00 ± 1.53 ^@^	52.33 ± 2.96 ^¥^	20.33 ± 2.60	5.67 ± 0.88
Extract 1.5 µg/mL	66.66 ± 1.67 ^¥^	67.00 ± 4.16 ^¥^	60.66 ± 1.76 ^¥^	18.66 ± 1.76 ^@^	3.33 ± 0.33 ^@^

Results were expressed as mean ± SEM (*n* = 3) and significance differences were compared related to the control group. ^@^ *p* <0.05 and ^¥^ *p* <0.001 by One Way ANOVA and Tukey post hoctest.

**Table 5:** Effect of *A. harveyi* leaf extract on viable, early apoptotic, apoptotic and necrotic sperm of bull frozen-thawed semen using the annexinV/PI assay.

Sample	Viable (%)	Early Apoptosis (%)	Apoptosis (%)	Necrosis (%)
	(A−/PI−)	(A+/PI−)	(A+/PI+)	(A−/PI+)
Untreated control	42.60 ± 2.19	2.65 ± 0.03	30.40 ± 1.56	24.50 ± 0.75
Extract 0.5 µg/mL	47.35 ± 1.01	2.20 ± 0.00 **	27.00 ± 0.92 *	23.45 ± 0.09
Extract 1.0 µg/mL	58.10 ± 0.23 ***	2.00 ± 0.00 ***	14.25 ± 0.14 ***	25.45 ± 0.49
Extract 1.5 µg/mL	62.65 ± 1.76 ***	1.80 ± 0.17 ***	10.50 ± 0.98 ***	25.05 ± 0.61

Results were expressed as mean ± SEM (*n* = 3) significance differences were compared related to the control groups. * *p* < 0.05, ** *p* < 0.01 and *** *p* < 0.001 by One Way ANOVA and Tukey post hoctest.
